# Preimplantation genetic testing for Cockayne syndrome with a novel ERCC6 variant in a Chinese family

**DOI:** 10.3389/fgene.2024.1435622

**Published:** 2024-10-15

**Authors:** Xuemei He, Yiyuan Zhang, Xianjing Huang, Pingping Qiu, Hong Ji, Lu Ding, Yingying Shi, Yanru Huang, Ping Li, Libin Mei

**Affiliations:** ^1^ Department of Reproductive Medicine, Women and Children’s Hospital, School of Medicine, Xiamen University, Xiamen, China; ^2^ Xiamen Key Laboratory of Reproduction and Genetics, Women and Children’s Hospital, School of Medicine, Xiamen University, Xiamen, China; ^3^ School of Life Sciences, Xiamen University, Xiamen, China; ^4^ Department of Central Laboratory, Women and Children’s Hospital, School of Medicine, Xiamen University, Xiamen, Fujian, China; ^5^ School of Public Health, Xiamen University, Xiamen, Fujian, China

**Keywords:** Cockayne syndrome, whole-exome sequencing, ERCC6, preimplantation genetic testing for monogenic disorders (PGT-M), embryo

## Abstract

**Background:**

Cockayne syndrome (CS) is a rare, multisystem, autosomal recessive disorder characterized by cachectic dwarfism, nervous system abnormalities, and premature aging. Mutations in the ERCC6 and ERCC8 genes are the predominant causes of Cockayne syndrome, with ERCC6 gene mutations present in approximately 75% of cases.

**Methods:**

Trio-based whole-exome sequencing (trio-WES) was employed to identify potential pathogenic variants associated with CS. Preimplantation genetic testing for monogenic disorders (PGT-M) was conducted to prevent the transmission of the pathogenic variant.

**Results:**

Two compound heterozygous mutations were identified in ERCC6—c.1297G>T (p. Glu433*) and c.1607T>G (p. Leu536Trp)—with c.1297G>T representing a novel mutation. Four blastocysts resulting from intracytoplasmic sperm injection were subjected to biopsy. Genetic analyses revealed that E1 harbored maternal mutations in diploid embryos, E2 and E3 carried both paternal and maternal mutations in non-diploid embryos, and E4 did not carry paternal or maternal mutations in diploid embryos. Following the transfer of the E4 embryos, a single successful pregnancy was achieved.

**Conclusion:**

The successful application of PGT-M in this family offers a potential approach for addressing other monogenic diseases. The findings of this study broaden the variant spectrum of ERCC6 and will contribute to the molecular diagnosis and genetic counseling of CS. This case highlights the feasibility and effectiveness of PGT-M in preventing CS and provides valuable insights for similarly affected families.

## 1 Introduction

Cockayne syndrome (CS) is a rare autosomal recessive disorder of the nervous system stemming from a deficiency in nucleotide excision repair (NER). Named after Dr. Cockayne’s initial report from the UK in 1936, it is characterized by growth disorders, intellectual disabilities, progressive neurological and cognitive decline, as well as impaired vision and hearing. Affected children may also present with cachexia and premature aging (Cheng et al., 2023; Vessoni et al., 2020). Incidence rates of CS in the United States, Western Europe, and Japan are approximately 2.5/100 million, 1/2.7 million, and 1/2.77 million (Karikkineth et al., 2017; Kubota et al., 2015), respectively. However, there are currently no reported statistics on incidence rates in China.

CS exhibits a high degree of genetic heterogeneity, primarily caused by mutations in five genes: ERCC8 (CSA), ERCC6 (CSB), ERCC5 (XPG), ERCC3 (XPB), and ERCC2 (XPD) (Xie et al., 2017; Araújo and Kuraoka, 2019). Among these, ERCC6 and ERCC8 are the main pathogenic genes, with ERCC6 mutations being the most prevalent, accounting for approximately 75% of cases. ERCC6 encodes the DNA excision repair protein ERCC6 (CSB), which belongs to the SWI2/SNF2 helicase family and plays roles in chromosomal remodeling, transcriptional regulation, and DNA repair (Liu et al., 2015; Dürr et al., 2005; Beerens et al., 2005). CS proteins are crucial for the transcription-coupled nucleotide excision repair (TC–NER) process, and deficiency in TC–NER due to pathogenic CSB variants can lead to various hereditary diseases (Tiwari et al., 2021; Saijo, 2013).

Preimplantation genetic testing (PGT-M) is extensively employed in in vitro fertilization (IVF) centers globally to mitigate the risk of monogenic diseases in embryos transferred to mothers and to enhance fertility outcomes. Here, we present a case involving a Chinese family with CS resulting from novel compound heterozygous pathogenic variants in the ERCC6 gene. Following genetic counseling, the couple chose PGT-M, resulting in a successful pregnancy.

## 2 Materials and methods

### 2.1 Case description

**FIGURE 1:**
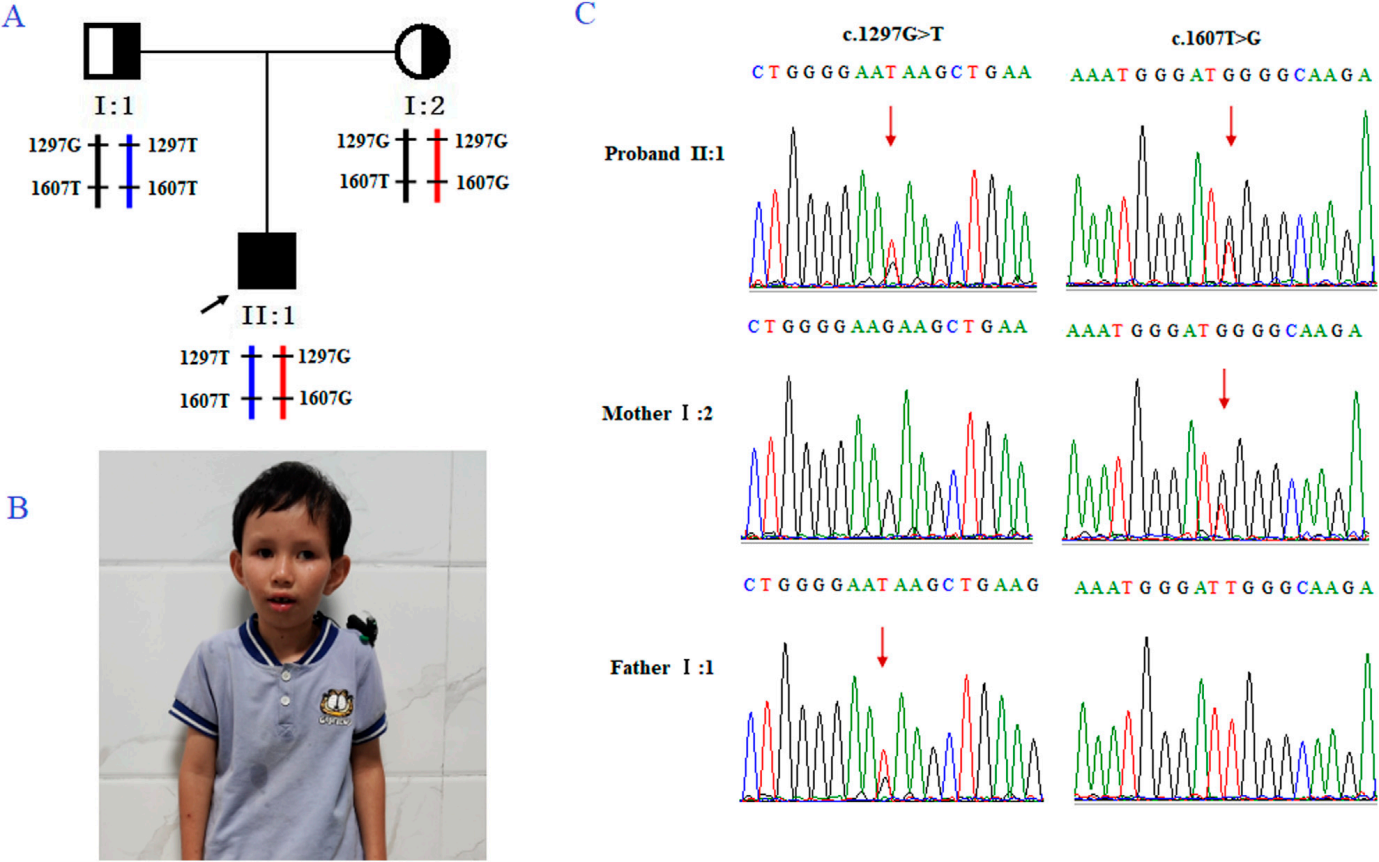
Genetic analysis of the case family. **(A)** Pedigree of the family with CS. The black arrow indicates the proband (II-2). **(B)** Clinical features of the proband, presenting with microcephaly and premature aging facial features. **(C)** Sanger sequencing of family members I-1, I-2, and II-2 (the proband). The red arrow highlights the variant site (c.1297G>T and c.1607T>G).

The family (Figure 1A) in this study was from Fujian Province, China. The proband (II2; Figure 1B was a male who visited an external hospital at the age of 2 years and 11 months. Clinical features included microcephaly, aging facial features, refractive errors, exotropia, moderate hearing impairment (53bB HL), delayed language development, intellectual impairment, spasmodic paraplegia, abnormalities, and valgus deformity. The parents had normal phenotypes and were not close relatives. The patient denied a family history of genetic diseases. After genetic counseling, the couple underwent genetic testing to determine the cause of the disease. To prevent the subsequent transmission of pathogenic mutations to the next-generation, the couple was counselled and recommended to receive PGT-M treatment. This study was approved by the internal ethics committee of Women and Children’s Hospital, Xiamen University, and informed consent was obtained from all participants.

### 2.2 Methods

#### 2.2.1 Molecular genetic analyses

Genomic DNA was extracted from peripheral blood leukocytes using a QIAamp Blood Kit (QIAGEN, Hilden, Germany) following the manufacturer’s protocol. The exons of DNA samples were captured using an AgilentSureSelect human whole exome V6 assay kit system (Agilent Technologies, Inc., Santa Clara, CA, United States) and subjected to high-throughput sequencing using a NovaSeq6000 sequencer (Illumine Inc., San Diego, California, United States). Sequencing reads were aligned to the UCSC human reference genome version hg19 using BWA. GATK and VarScan were used to screen for single-nucleotide polymorphism (SNP) and insertion and deletion mutations in the sequence. After using 1,000 Genomes, the dbSNP database, ESP6500, ExAC, and an in house database for filtering and screening, the mutation frequency was < 0.01 mutation sites. Software including Polyphen-2, SIFT, and MutationTaster was used to predict the pathogenicity of suspicious mutations. Sequence mutation sites were interpreted according to the “Sequence Variation Interpretation Standards and Guidelines” published by the American Society of Medical Genetics and Genomics. Finally, harmful mutations related to the phenotype of the proband were identified.

#### 2.2.2 IVF and blastomere biopsy

Using an antagonist regimen for ovulation induction, fertilization was completed using intracytoplasmic sperm microinjection (ICSI). Gardner’s blastocyst morphology scoring method was used to select blastocysts with a gestational age of ≥ 3BC at D5 or D6 days for trophoblast biopsy. Approximately 5–8 trophoblast cells were taken, and live cells were transferred to blastocysts containing 5 μL dedicated collection tube for L lysis solution. The blastocysts after biopsy were cryopreserved by vitrification. Whole genome amplification (WGA) was performed using multiple annealing and looping-based amplification cycles (MALBAC).

#### 2.2.3 PGT with haplotype and copy number variation (CNV) analyses

The WGA products of each embryo were subjected to Sanger sequencing to directly identify the mutations. Owing to the limited number of cells available for amplification, it was difficult to avoid allele deletion (ADO); to prevent misdiagnosis, haplotype analysis was conducted using SNP markers Using Illumina’s Asian Screening Array (ASA) gene chip, and SNP sites on chromosome 10, where the ERCC6 gene is located, were detected. Within a range of 2 Mb upstream and downstream of WNT10B, effective SNP sites that could distinguish between high- and low-risk haplotypes were selected for subsequent embryo testing. In addition to the detection of ERCC6 mutations, CNV analysis was performed using WGA products to prevent embryonic abortion, death, or other problems that may be caused by embryonic chromosomal abnormalities. Deletions or duplications of > 4 Mbp and mosaicism (larger than 10 Mb) of > 30% were reported in each embryo.

#### 2.2.4 Embryo transfer and prenatal diagnosis

Embryo selection for transfer was determined based on the morphological scores and PGT results. Clinical pregnancy was defined as 28 days after frozen embryo transfer (FET), when fetal cardiac pulsation was detected on ultrasound examination. To verify the diagnosis of PGT, Sanger sequencing, chromosomal karyotype analysis, and CNV sequencing were performed on fetal DNA obtained via amniocentesis at 18 weeks of gestation.

## 3 Results

### 3.1 Genetic analysis

**FIGURE 2:**
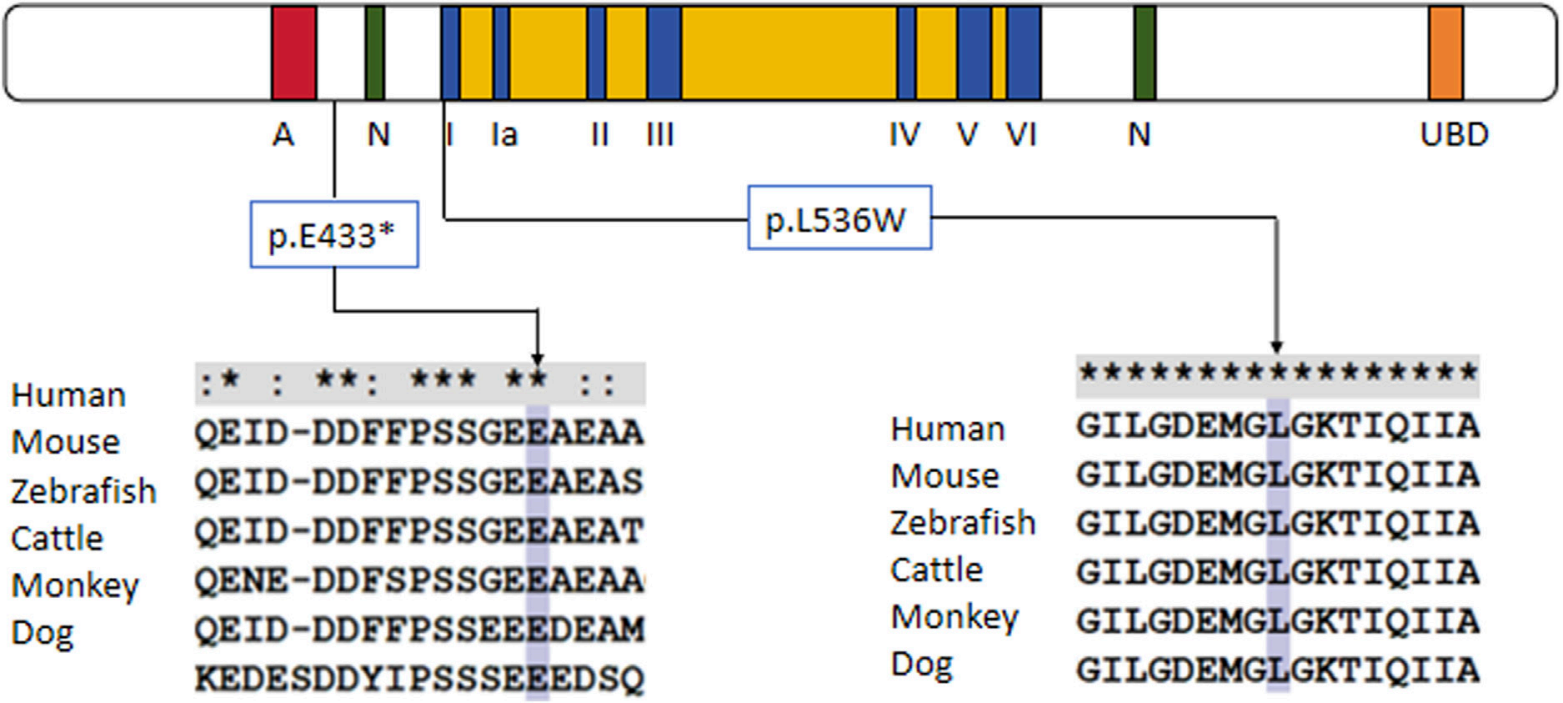
Protein alignment demonstrates the conservation of ERCC6 p.E433X and p.L536W residues across multiple species, indicating that the two mutations occurred at evolutionarily conserved amino acids.

Trio-based whole-exome sequencing (trio-WES) analysis identified a compound heterozygous mutation (NM_000124.4: c.1297G>T (p.Glu433Ter) and c.1607T>G (p.Leu536Trp) in the ERCC6 gene of the proband, which was inherited from both parents (Figure 1C). The c. 1297G > T variant is a nonsense mutation in exon 5 of ERCC6 and is predicted to introduce a premature stop codon (p.E433X), which is classified as a likely pathogenic mutation according to the American College of Medical Genetics and Genomics (ACMG) guidelines (PVS1, PM2). This mutation has not been previously reported and was not found in HGMD, ClinVar, ExAC,ESP6500, dbSNP, or any other SNP databases. The variant c.1607T>G is a missense mutation in exon 7 of ERCC6 that results in a leucine substitution for tryptophan at amino acid 536 (p.L536W), which is classified as a pathogenic mutation according to the American College of Medical Genetics and Genomics (ACMG) guidelines (PM2, PP3, PP1, PM3). ClustalX sequence alignment revealed that the regions containing p.E433X and p.L536W are highly conserved across various species (Figure 2).

### 3.2 Embryo preparation and genetic testing

**FIGURE 3:**
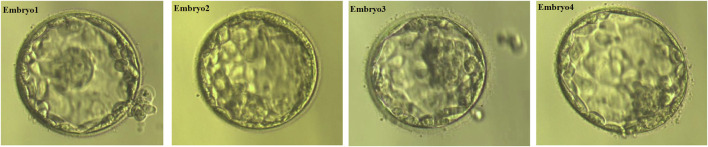
Growth and development of four embryos.

**FIGURE 4:**
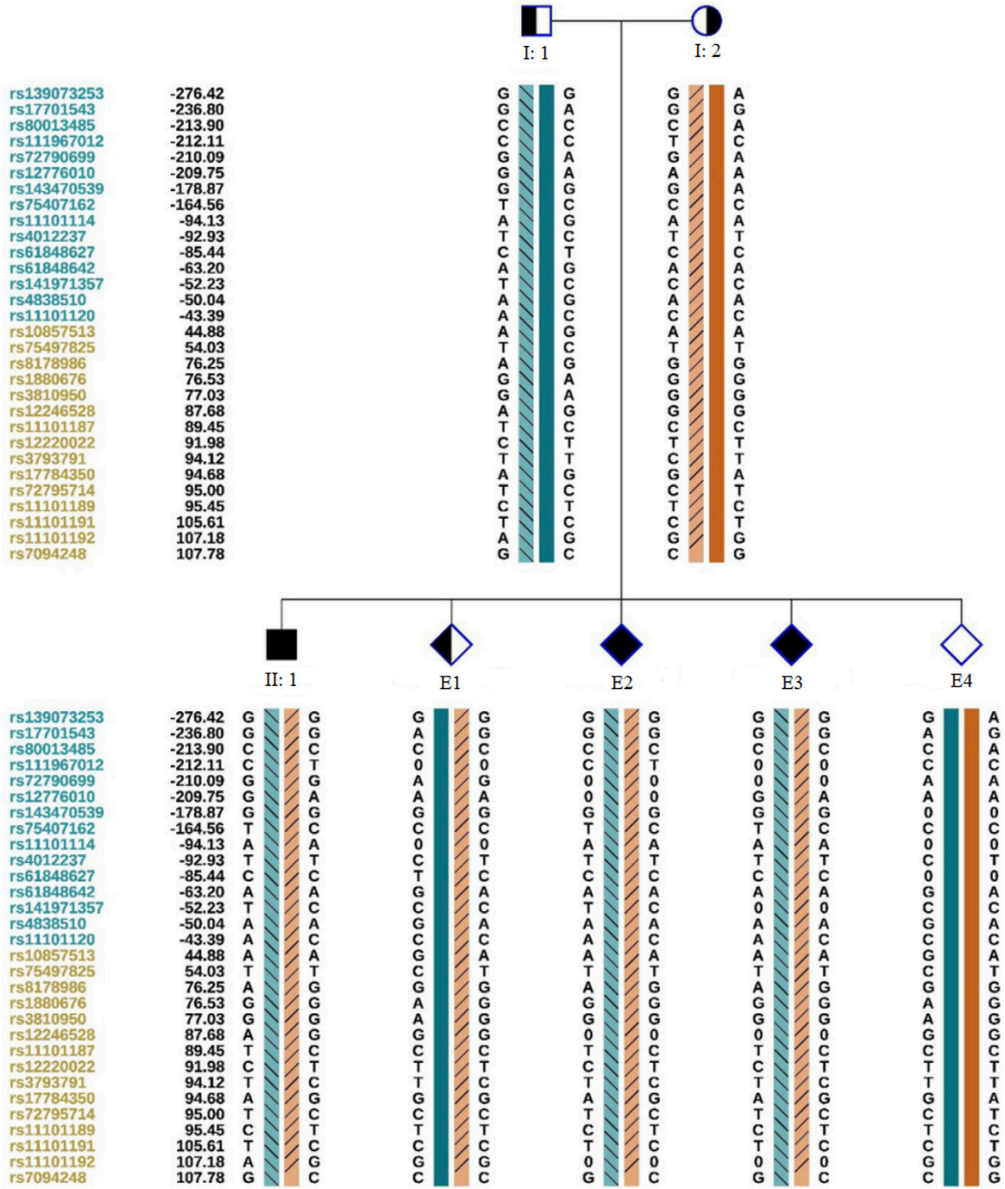
Schematic representation of SNP-based haplotypes. SNP sites are indicated on the left, with blue and orange representing SNP sites upstream or downstream, respectively, of the chromosomal position where the gene is located. Light orange and light blue left slashes denote high-risk haplotypes, while dark blue bars represent normal or low-risk haplotypes of the father, and dark orange bars represent normal or low-risk haplotypes of the mother. E1 inherited the maternal haplotype, E2 and E3 inherited both maternal and paternal haplotypes, and E4 possessed a normal haplotype.

We obtained 10 oocytes under ultrasonographic guidance after the induction of ovulation. Among the 10 embryos fertilized by ICSI, four (E1, E2, E3,E4; Figure 3) were suitable for biopsy. We successfully amplified the DNA of the blastocyst by MALBAC. CNV results showed that two embryos (E1 and E4) had a normal karyotype. The other two embryos (E2 and E3) exhibited chromosomal abnormalities. Sanger sequencing showed that E1 carried the c.1607T>G mutation, and both E2 and E3 carried the c.1297G>T and c.1607T>G mutations; no mutation was found in E4. To verify the Sanger sequencing results, we selected SNPs that could be used for the determination of the high- and low-risk haplotypes after pre-testing to infer the disease status of the embryos. SNP-based haplotyping showed that E1 inherited the maternal high-risk chromosome carrying the c.1607T>G mutation, E2 and E3 inherited both the maternal high-risk chromosome and paternal high-risk chromosome, whereas E4 carried neither mutation, which was consistent with the Sanger sequencing results (Figure 4; Table 1).

**TABLE 1:** Genetic screening results of embryos.

Embryo	Biopsy time	Gardner grade^a^	Copy number variations	SNP haplotyping^b^	Sanger sequencing
E1	D5	4BB	46, XN	F-Hap B/M-Hap A	c.1607T>G
E2	D5	4BC	−4	F-Hap A/M-Hap A	c.1297G>T/c.1607T>G
E3	D5	4BC	+21	F-Hap A/M-Hap A	c.1297G>T/c.1607T>G
E4	D5	4BC	46, XN	F-Hap B/M-Hap B	normal

^a^ The embryo quality was assessed following the Gardner grading system.

^b^ F-Hap A: haplotype A of the father,c.1297G>T; F-Hap B: haplotype B of the father, wild-type; M-Hap A: haplotype A of the mother, c.1607T>G; M-Hap B: haplotype B of the mother, wild-type.

### 3.3 Prenatal diagnosis and pregnancy outcome

**FIGURE 5:**
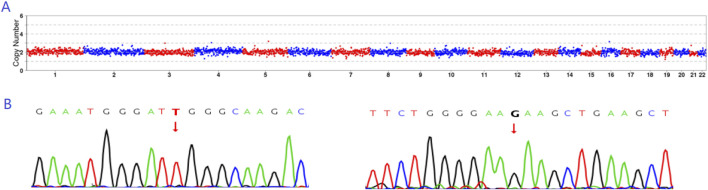
Prenatal diagnosis results. **(A)** CNV results indicating that E4 exhibited euploidy. **(B)** Sanger sequencing results revealing that E4 did not carry either the c.1297G>T or c.1607T>G mutation.

Based on embryo selection principles, the euploid embryo E4, which did not carry a ERCC6 mutation, was transferred into the uterus. On the 13th day after transplantation, the serum level of human chorionic gonadotropin was 8,283 mIU/mL. Ultrasound examination on the 28th day indicated a single live fetus in the uterus. The prenatal diagnosis results were consistent with those of PGT-M (Figures 5A, B).

## 4 Discussion

CS is also known as cocaine syndrome, dwarfism, retinal atrophy, deafness syndrome, cerebellar and striatocerebellar calcification, and white matter malnutrition syndrome (Epanchintsev et al., 2020). Patients often experience growth retardation, developmental delay, and special manifestations, including microcephaly, sunken eyeballs, abnormal skin photosensitivity, progressive sensorineural hearing loss, progressive retinal pigment degeneration, accumulation in the liver, kidney, cardiovascular system, and endocrine function (Wu et al., 2019). Most patients die before the age of 20, often from respiratory or renal failure caused by cachexia (Kou et al., 2018).

ERCC6 is located on chromosome 10 (10q11.3) and encodes a 1,493 amino acid CSB protein with 168 kDa. It is a member of the SWI2/SNF2 family and includes two important functional domains, DEXDc and HELICc, which play roles in chromatin maintenance and remodeling (Lee et al., 2019; Wang et al., 2022). ERCC6 is involved in the transcription of oxidative DNA damage, DNA base excision repair (BER), and transcriptionally coupled nucleotide excision repair (in UV-induced DNA damage) (Okur et al., 2020). ERCC6 mutations lead to transcriptionally coupled nucleotide repair (TC–NER) defects, and there are also reports that ERCC6 mutations may cause mitochondrial dysfunction, protein synthesis disorders, and loss of protein stability (Qiang et al., 2021). These functional deficiencies can cause children with CS to exhibit symptoms such as skin photosensitivity, neurodegenerative changes, premature aging, developmental delay, and short stature.

The c. 1297G > T (p. Glu433*) mutation is a novel nonsense mutation that has not been reported previously. The mutation caused the CBS protein to introduce a termination codon at amino acid 433, leading to the interruption of the CBS protein and loss of protein function. Previous studies have reported that CBS mutations lead to a higher proportion of nonsense and splicing mutations during protein truncation. Kou et al. found that truncation mutations in CBS lead to more severe CS phenotypes such as developmental and neurological abnormalities (Kou et al., 2018). The damage to the N-terminal domain of CSB protein impairs its chromatin remodeling activity, specifically by preventing the eviction of histones from chromatin at double-strand breaks (DSBs) (Batenburg et al., 2017). In this study, the CSB protein is truncated, retaining only part of the N-terminus while losing a significant portion of the sequence extending from the N-terminus to the C-terminus. As a result, histones may accumulate around the DSBs, failing to be effectively evicted. This accumulation can lead to a tighter chromatin structure, thus restricting the accessibility and function of DNA repair factors. Consequently, DNA repair efficiency may decline, increasing genomic instability and enhancing cellular sensitivity to DNA damage. Zhang also reported a similar case, identifying a typical CS patient with both a c.1387C>T (p.Q463*) mutation in the ERCC6 gene and a 2.82 Mb microdeletion (Zhang et al., 2011). This case exemplifies the typical CS phenotype caused by only a truncated N-terminal CSB protein.

The c. 1607T > G (p. Leu536Trp) mutation is a missense mutation in exon 7. This mutation was mentioned in Vessoni (Vessoni et al., 2020) and was later reported in detail by Yu et al. (2014) and Zhou et al. (2016) in Chinese families, both of which were typical cases of CS. The CSB protein contains an ATP-binding domain between residues 519 and 695; the c.1607T>G mutation site is located in this domain, causing abnormal ATP hydrolysis in the CBS protein and affecting the nucleotide cleavage pathway (NER), thereby affecting the role of ERCC6 in DNA repair.

Currently, there is no effective treatment for CS in clinical practice, and symptomatic treatment is the main approach (Wang et al., 2019; Mahindran et al., 2021; Cao et al., 2024) CS has an autosomal recessive inheritance pattern. Patients with CS often have serious clinical manifestations and poor social participation, imposing a serious burden to their families and society. PGT is based on genetic analysis of individual cells using high-throughput sequencing technology. It can be used to screen for aneuploidy across the entire genome, but can also detect single nucleotide variations, avoiding the implantation of genetically defective embryos from the source. Previous study has reported two successful cases of PGT-M intervention in CSA type due to mutations in the ERCC8 gene, emphasizing the efficacy of PGT-M in treating hereditary neurological diseases or metabolic disorders with nervous system phenotypes (Zou et al., 2024).In this study, after receiving detailed genetic counseling from the parents of the proband, genetic disease-related testing was performed on blastocysts implanted in the mother’s body using PGT-M technology. Two transferable embryos were successfully selected and priority was given to the transfer of haploid embryos that did not carry paternal or maternal mutations to achieve a single pregnancy. Therefore, it is of great clinical significance to carry out genetic diagnosis before embryo implantation to achieve early prevention and intervention and avoid the birth of CS patients.

## 5 Conclusion

In summary, we report a case of a family with CS caused by an ERCC6 mutation. Through WGA, NGS-based haplotype analysis, and prenatal diagnosis, to reduce the risk of misdiagnosis from PGT-M, the couple finally gave birth to a healthy baby. Our results confirm the feasibility of PGT-M in blocking intergenerational transmission of the ERCC6 mutation, which has important clinical applications in the prevention and control of birth defects.

## Data Availability

The datasets presented in this study can be found in online repositories. The names of the repository/repositories and accession number(s) can be found in the article/supplementary material.
